# Multiple Oligo assisted RNA Pulldown via Hybridization followed by Mass Spectrometry (MORPH-MS) for exploring the RNA-Protein interactions

**DOI:** 10.1080/15476286.2023.2287302

**Published:** 2023-12-17

**Authors:** Priyanka Pant, Regalla Kumarswamy

**Affiliations:** a CSIR-Centre for Cellular and Molecular Biology, Uppal Road, Hyderabad, India; b Academy of Scientific and Innovative Research (AcSIR), Ghaziabad, Uttar Pradesh, India

**Keywords:** RNA-pulldown, long non-coding RNA, RNA-protein interaction, ASO pulldown, affinity purification-mass spectrometry (AP-MS)

## Abstract

Understanding RNA-protein interactions is crucial for deciphering the cellular functions and molecular mechanisms of regulatory RNAs. Consequently, there is a constant need to develop innovative and cost-effective methods to uncover such interactions. We developed a simple and cost-effective technique called Multiple Oligo assisted RNA Pulldown via Hybridization (MORPH) to identify proteins interacting with a specific RNA. MORPH employs a tiling array of antisense oligos (ASOs) to efficiently capture the RNA of interest along with proteins associated with it. Unlike existing techniques that rely on multiple individually biotinylated oligos spanning the entire RNA length, MORPH stands out by utilizing a single biotinylated oligo to capture all the ASOs. To evaluate MORPH’s efficacy, we applied this technique combined with mass spectrometry to identify proteins interacting with lncRNA NEAT1, which has previously been studied using various methods. Our results demonstrate that despite being a simple and inexpensive procedure, MORPH performs on par with existing methods.

**Abbreviations**: ASO, Antisense oligo; lncRNA, long non-coding RNA; MORPH, Multiple Oligo assisted RNA Pulldown via Hybridization

## Graphical abstract

**Figure:**
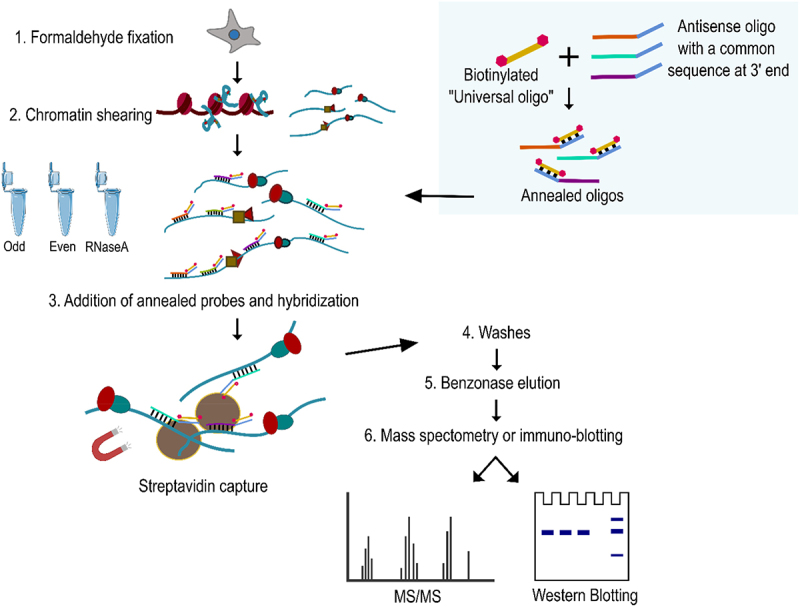

## Introduction

RNA is found rarely in isolation inside cells. It typically acts in conjunction with associated proteins, which often dictate its function. Due to advancements in deep sequencing, several thousands of long non-coding RNAs (lncRNAs) have been identified which may serve structural or regulatory roles. The proteins they interact with frequently define regulatory functions. For example, the X chromosome-associated lncRNA *XIST* recruits the histone deacetylase, HDAC complex subunit split end family protein (SPEN) to chromosome X, resulting in its transcriptional inactivation [1]. Telomeric RNAs (*TERRA*), transcribed from sub-telomeric regions, play a role in maintaining genome stability. TERRA binds to ATRX and prevents its binding to the telomere, thereby assisting in maintaining telomere length [2]. Similarly, the cytoplasmic lncRNA Noncoding RNA activated by DNA damage (*NORAD)* sequesters Pumillio, preventing genome instability [3], and the cytoplasmic lncRNA Noncoding repressor of Nuclear Factor of Activated T cells (*NRON)* hinders the shuttling of NFAT from the cytoplasm to the nucleus [4] by forming a RNA-protein complex.

Several methods have been described to detect RNA-protein interactions with approaches focusing on either the RNA or protein component. Protein-centric methods typically involve the use of antibodies to capture the interacting RNA. On the other hand, RNA-centric methods involve RNA enrichment followed by mass spectrometry (MS) for protein detection. For instance, *in-vitro* transcribed RNA can be incubated with cell lysate and subsequently analysed by MS/MS [5]. This approach can predict the interactors albeit with low confidence because it is not conducted inside living cells and a lack of post-transcriptional RNA modifications can give rise to false interactors. Alternatively, other protocols such as RNA antisense purification (RAP-MS) [6], capture hybridization analysis of RNA targets (CHART) [7], chromatin isolation by RNA purification (ChIRP-MS) [8] and hybridization purification of RNA−protein complexes followed by mass spectrometry (HyPR-MS) [9] pull down the RNA of interest from crosslinked cells using a biotinylated ASOs. These methods enable the isolation of interacting RNA, DNA, or proteins, as interactions are preserved by crosslinking. One of the drawbacks of such protocols is that they require multiple individually biotinylated probes which increases the cost of probe synthesis making the overall process expensive. In this manuscript, we introduce MORPH - Multiple Oligo assisted RNA Pulldown via Hybridization, a technique that addresses the cost issue associated with probe synthesis. MORPH utilizes a single biotinylated ‘Universal oligo’ to perform RNA pulldowns. Inspired by the smiFISH protocol [10], the complementary tiling oligos in MORPH contain a shared binding sequence at their 3’ end. As proof of principle, we used MORPH-MS for exploring proteins interactors of lncRNA NEAT1. By comparing NEAT1 interacting proteome data from MORPH with data from a previously published study, we show that MORPH performs on par with existing RNA capture-based methods.

## Materials and methods

### Design of antisense oligos

Complementary probes were designed using the ChIRP probe designer tool from LGC Biosearch (https://www.biosearchtech.com/support/tools/design-software/chirp-probe-designer), with a masking level of 5. The tiling oligos were prepared to get 1 oligo/350–450 RNA length. The GC content of the complementary region was maintained around 48–52%. To ensure specificity, all the oligos were manually screened for non-specific binding using NCBI blast, and only probes with an E-value below 0.0002 were chosen. Additionally, a common binding sequence 5’-TTACACTCGGACCTCGTCGACATGCATT-3’ was added towards the 3’ end of all oligos (Table S1, Supplementary Fig. S1). The MORPH probes were desalted and (Table S1) were ordered from Bioserve, India Pvt. Ltd. The biotinylated universal oligo, which had TEG-biotin attached to both the 5’ and 3’ ends, was designed to be complementary to the common sequence was HPLC purified and ordered from GCC Biotech, India.

### Probe annealing

All the oligos were dissolved in Tris-EDTA pH 8.0 (TE) buffer to a final concentration of 200 μM. The complementary probes were divided into two sets: Odd and Even, based on their order of placement on the RNA. To achieve an equimolar concentration of all the complementary probes, 20 μL from each probe set were pooled together, resulting in a final concentration of 200 μM. For annealing, 25 μL of the Universal oligo and 25 μL of the pooled oligos were combined in a tube, along with 5 μL of 10× NEB buffer 3.1. The tube was then placed in a beaker filled with hot water (80–85°C) and allowed to cool down gradually. The successful annealing of the probes was confirmed by resolving them on a 6% polyacrylamide gel electrophoresis (PAGE) gel prepared in Tris-borate-EDTA (TBE) buffer. The gel is stained using Ethidium Bromide.

### Collection of cells and chromatin shearing

AC16 cells were obtained from Millipore and cultured in DMEM/F12 medium (Gibco,12500062) supplemented with 12.5% foetal bovine serum (Gibco 16,000,069). Approximately 20 million cells were used for each pull-down group, including the odd, even, and RNaseA-treated groups. To initiate the cross-linking process, the cell pellets were resuspended in 3% paraformaldehyde in PBS and incubated on an end-to-end rotator for 30 min. The cross-linking reaction was then stopped by adding 125 mM glycine and incubating for an additional 10 min. The cell suspension was subsequently washed twice with PBS, and the resulting pellet was snap-frozen and stored at −80°C. For chromatin shearing, the cross-linked cell pellets were resuspended in lysis buffer (50 mM Tris-HCl, 10 mM EDTA, 1% SDS, 1 mM PMSF and RNase inhibitor) at a ratio of 1 ml per 100 mg of pellet weight. The suspension was transferred to 1.5 ml Diagenode tubes and subjected to sonication using the Bioruptor Pico (Diagenode). Sonication was performed for 15 min, with 30 s of sonication followed by 30 s of rest, until the solution became clear. The sonicated sample was then centrifuged at 16,000 g for 10 min at 4°C to remove any debris, and the clear supernatant was pooled into a single tube for further analysis.

### Bead pre-clearing and hybridization

To perform pre-clearing, 15 µL of Pierce™ Streptavidin Magnetic Beads (#88817) per sample were washed three times with lysis buffer. The sheared lysate was divided equally into three tubes, and 15 µl of washed beads was added to each tube. In the RNaseA negative control tube, 1 µg/ml of RNaseA (#R6148, Sigma Aldrich) was included. All the samples were incubated for preclearing in a hybridization oven at 37°C for 30–60 min with constant slow rotation. After this step, the precleared samples (2 ml/tube) were transferred to fresh 15 ml tubes, and twice the volume of freshly prepared hybridization buffer (750 mM NaCl, 1% SDS, 50 mM Tris-HCl pH 7.0, 1 mM EDTA, 15% Formamide, 1 mM PMSF, PIC, and RNase inhibitor.) was added to each tube. Subsequently, 100 pmol of annealed oligos were added to the corresponding tubes. In the RNaseA tube, 100 pmol of both Odd and Even probes were added. The tubes were sealed with parafilm and placed in a hybridization oven for 16 h at 37°C with end-to-end rotation to allow for hybridization to occur.

### Capture and wash

Just prior to the completion of hybridization, the beads (100 µl beads per pulldown) were washed twice with the lysis buffer on a magnetic stand. The washed beads were then added to separate 15 ml tubes corresponding to each pull-down group. The tubes were placed in a hybridization oven at 37°C with end-to-end rotation for 2 h. Following the incubation, the tubes were placed on a size-appropriate magnetic stand, and the beads were transferred to 1.5 ml tubes to facilitate the washing process. For stringent washes, the beads were washed with 1 ml of wash buffer (2× Saline-sodium citrate (SSC); diluted from 20× SSC, Invitrogen #AM9763, 0.5% SDS, 1 mM PMSF freshly added) by keeping the tubes in the hybridization oven at 37°C with end-to-end rotation for 5 min. This washing step was repeated 3–5 times to ensure thorough washing of the beads. To check the efficiency of the pulldown, 1% volume of the beads from all groups was separated for further analysis.

### RNA isolation

For RNA isolation, 100 µL of PK buffer (100 mM NaCl, 10 mM Tris – HCl pH 7.0, 1 mM EDTA, 0.5% SDS, 2ul ProteinaseK (VWR #0706) from 20 mg/mL stock) were added to the 1% beads and ‘INPUT’ samples. The mixture was incubated at 37°C for 1 h. Following the incubation, the samples were heated at 75°C for 10 min. Then, 500 µL of TRIzol (RNA Iso plus, Takara #9108) was added, and RNA isolation was performed according to the manufacturer’s instructions. The resulting RNA pellet was resuspended in nuclease-free water and the RNA samples were treated with DNaseI. The DNA-free RNA samples were then used for cDNA synthesis using the Primescript cDNA synthesis (Takara #6110A), following the manufacturer’s instructions. For quantitative PCR (qPCR), NEAT1-specific primers (Table S4) were used in combination with the SYBR green master mix (Takara #RR820).

### Western blot of pulldown samples

10% of the beads were collected and boiled in SDS buffer for 10 min. The samples were then loaded onto a 4–12% Tris gradient gel in MES buffer (100 mM MES, 100 mM Tris – HCl, 2 mM EDTA, 7 mM SDS). Immunoblotting was performed using an anti-SFPQ antibody (Proteintech 15,585–1-AP), anti-COAA (Bethyl labs, A300–845-T), anti-HnRNPK (Santa cruz, sc -28,380), or anti-RALY (Bethyl labs, A302-069A-T), at a dilution of 1:1000 and Anti rabbit HRP secondary antibody (Abcam, ab7090) at a dilution of 1:10,000.

### Mass spectrometry of pulldown samples

The Pierce magnetic beads were incubated with 200 µL of nuclease elution buffer (20 mM Tris – HCl pH 8.0, 0.125% N-lauroylsarcosine, 2 mM MgCl_2_, 10 mM DTT, 125 U/ml Benzonase Sigma- # 707463) and kept at 37°C for 30 min. This step was repeated. The pooled supernatant (400 µL) was concentrated using a 3 kDa MWCO filter until the volume reached 30 µL. The samples were then boiled after adding SDS loading dye for 30 min to reverse crosslinking and loaded onto a 4–12% Tris gradient gel in MES buffer (100 mM MES, 100 mM Tris – HCl, 2 mM EDTA, 7 mM SDS) at 200 V for 5 min, fixed in 50% Methanol and 5% acetic acid and stained with Coomassie brilliant blue. Each lane of the gel was cut into two strips based on higher (above 75 kDa) and lower molecular weight (below 75 kDa) regions. The gel pieces from each lane were diced into approximately 1–2 mm^2^ pieces and washed twice with a wash buffer (25 mM NH_4_HCO_3_, 50% acetonitrile) to remove the Coomassie stain completely. The gel pieces were dehydrated with acetonitrile and dried completely using a speed-vac. For reduction, the gel pieces were incubated in 10 mM DTT prepared in 50 mM NH_4_HCO_3_ at 56°C for 45 min. The liquid was replaced with freshly prepared 55 mM iodoacetamide in 50 mM NH_4_HCO_3_ for alkylation, and the tubes were incubated at room temperature for 30 min [11]. Gel pieces were washed with 50 mM NH_4_HCO_3_ for 10 min, followed by dehydration with acetonitrile. Next, the dried gel pieces were treated with 10 ng/µL trypsin (Promega gold) prepared in 25 mM NH_4_HCO_3_ and 1 mM CaCl_2_. The tubes were incubated at 37°C for 16 h for digestion. After 16 h, the peptides were extracted by subjecting the gel pieces to two rounds of extraction with 5% formic acid and 30% acetonitrile. The supernatant from both rounds of extraction was collected in a fresh tube and dehydrated using a Speed-Vac. The pellet was resuspended in 5% acetonitrile and 0.1% Trifluoroacetic acid. Desalting was performed using Pierce C18 Spin Tips, and the peptides were eluted in 50% acetonitrile and 0.1% Trifluoroacetic acid [12]. The spectra were collected on a Thermo Q Exactive and analysed using proteome discoverer software using sequest HT search engine with percolator validation against Uniprot database of Homo sapiens (release 2020.01) and database of known contaminants. Results were filtered for high confident peptides, with enhanced peptide and protein annotations. Before quantification, the normalization was done by total peptide amount with FDR of 0.01. Maximum allowed fold change was kept at 100.

### Silver staining

Equal volume of boiled lysate form Odd, Even, RNaseA, or lacZ was loaded on the gel along with protein ladder and 3% input and resolved. Once resolved, the gel was transferred to a glass dish and was fixed with fixative solution (50% methanol, 10% acetic acid, 50 μl formaldehyde) for 1 h. Post fixation, gel was washed twice with 50% ethanol for 20 min per wash and gel was treated with 20 mg/100 ml Sodium thiosulphate for precisely 1 min. Gel was then washed thrice with water 20 s per wash and incubated in silver nitrate solution (2 mg/ml) for 30 min. Silver nitrate solution was discarded, and gel was washed with water thrice 20 s per wash. The gel was incubated with developing solution (6% sodium carbonate, 0.05% formaldehyde, 0.0004% sodium thiosulphate) with continuous monitoring. Upon visibility of desired bands were visible, the reaction was stopped with 5% acetic acid.

### RNA-IP

2 × 10^6 cells (AC16 cells) were harvested and crosslinked with 1% formaldehyde in PBS for 10 min on an end-to-end rotor at room temperature. RNA-IP was performed as described earlier [13] using anti-SFPQ (Proteintech 15,585–1-AP), anti-COAA (Bethyl labs, A300–845-T), anti-HnRNPK (Santa cruz, sc -28,380), or anti-RALY (Bethyl labs, A302-069A-T). Anti-rabbit IgG (Abcam, ab171870) and anti-mouse IgG (Abcam, ab18443) were used as the negative controls. After the elution, RNA was dissolved in Nuclease free water and cDNA synthesis was done as described previously.

### Cloning and transfection

The full-length FUBP1 and NCL genes were isolated from AC16 cell line cDNA by employing the specified primers (Table S5). Subsequently, the resultant amplified DNA fragments were integrated into the pEGFP-C1 vector. The sequences of the inserted fragments were verified through Sanger DNA sequencing. Following this, 6 µg of the plasmids (pEGFP-FUBP1 and pEGFP-NCL) were introduced into HEK293T cells via transfection using PEI. The cells were collected and fixed 48 h after the transfection, in preparation for RNA immunoprecipitation (RNA-IP) using antibody against GFP (#11814460001, Roche). Mouse IgG was sued as the negative control..

### GO analysis and venn diagram

Gene ontology of the high confidence NEAT1 interactors is performed by Shiny GO [14] and the graph in Figure 2B was created using SR Plot. Preparation of Venn diagram and Fisher exact test were done using EVenn [15].

### PPI network analysis

The Uniprot IDs of 121 high-confidence NEAT1 interactors were utilized as input to construct a protein–protein interaction network through the STRING database (https://string-db.org/). To ensure the highest level of reliability, only the physical subnetwork was chosen to reveal the interactions among proteins that constitute a physical complex.

### Results and discussion

MORPH is an RNA-centric technique designed to identify the proteins interacting with a specific RNA of interest, it evades the need of using antibodies for probing RNA-protein interactions. This protocol can be used for RNAs of any length, whether they are cytoplasmic or chromatin-bound and can be used with various tissue types or cultured cells. Current state-of-the-art RNA pulldown methods involve the use of TEG-biotin-labelled ASO on fixed cell lysates. Such methods incur a huge cost due to use of several individually biotinylated oligos to capture the RNA of interest, and this cost would further increase if a tiling array of biotinylated probes are to be used for a longer RNA. Similar to the common binding sequence from the smiFISH technique [10], MORPH requires complementary antisense probes with a common sequence at the 3’ end of all the oligos and the same sequence has been used[10]. In this study, tilling array of ASOs against lncRNA NEAT1 with a common sequence at the 3’ end were designed (Supplementary Fig. S1, Table S1). This shared sequence enables the use of a common biotinylated ‘Universal oligo’ to capture all oligos. Prior to performing RNA pulldown from fixed cell lysates, the ‘Universal oligo’ and complementary oligos with a common 3’ sequence are annealed (Supplementary Fig. S2). These annealed oligos are then added to the cell lysate to allow hybridization with NEAT1.

### NEAT1 interacting proteome

**Figure 1.**
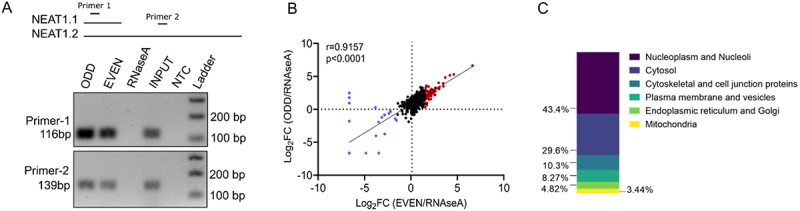
NEAT1 pulldown using MORPH (A) semi quantitative PCR using NEAT1 primers to assess enrichment of NEAT1 after MORPH-based pulldown in even, odd and RNaseA groups. Schematic indicate the relative position of primer binding (B) correlation plot of the Log_2_FC of the proteins detected in the ODD and EVEN set of oligos. (Pearson’s correlation of 0.91). (C) subcellular distribution of the proteins detected by MORPH-MS.

In this study, we employed the MORPH technique to perform a NEAT1 RNA pulldown. The tiling probes were divided into two sets: even and odd. We kept two controls where we performed pull down using a non-target oligo against LacZ. In order to eliminate the non-specific binding via the NEAT1 specific oligos, we also depleted RNA using RNaseA. We observed an enrichment of NEAT1 in ODD and EVEN set of probes (Figure 1A).

The samples were then subjected to mass spectrometry analysis. To verify the accuracy of MORPH, we conducted a correlation analysis of the Log2 fold change (Log2FC) values between the odd and even groups, resulting in a Pearson’s correlation coefficient of 0.91 (Figure 1B). We computed the log2FC based on the normalized abundance ratio of the odd or even group relative to the RNaseA treatment group. To ensure stringent analysis, only the proteins detected in both groups were considered. In total, we identified 148 proteins as NEAT1 interactors through mass spectrometry analysis. To evaluate the consistency of our methodology, we conducted a repetition of the experiment. Remarkably, out of the 148 proteins detected in the first experiment (experiment 1), 121 proteins were successfully identified when the experiment was repeated (experiment 2) (Table S2), confirming them as high-confidence NEAT1 interactors.

Similarly, when we normalized abundance ratios to the LacZ control, we identified a list of 32 NEAT1 interactors, 31 of which were also present in the dataset computed after normalizing with the RNaseA control. LncRNA NEAT1 is encoded as two isoforms – a 3.7kb NEAT1.1 and a longer 23kb isoform called NEAT1.2 and both the isoforms are associated with chromatin [16,17] and consistent with this, majority of the interacting proteins detected in this study are localized to the nucleoplasm or nucleoli (43.4%) (Figure 1C). NEAT1.1 isoform can localize to the cytoplasm under certain conditions [18]. We successfully detected previously reported cytoplasmic interactors of NEAT1.1, including alpha-enolase (ENO) with Log2FC of 2.7 (experiment 1) and 1.5 (experiment 2), and phosphoglycerate kinase (PGK) with Log2FC of −3.97 (experiment 1) and −1.61 (experiment 2) (Table S2) [18].

**Figure 2.**
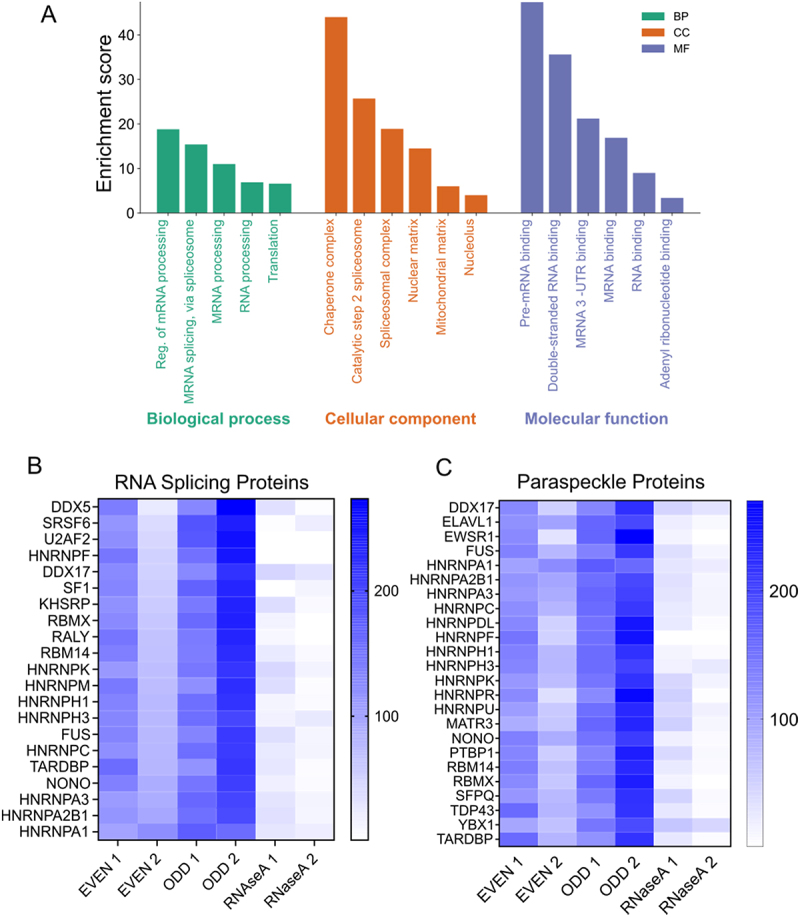
Gene ontology of the NEAT1 interacting proteins (A) bar graph depicting the enrichment score of the mentioned GO terms under biological processes (BP), Cellular component (CC) and Molecular function (MF) category. (B) heatmap showing the enrichment of the RNA splicing proteins detected in the ODD and EVEN pulldowns (C) heatmap showing the enrichment of the paraspeckles proteins in the ODD and EVEN sets of pulldown.

Gene ontology analysis of the NEAT1 interacting proteins revealed their involvement in RNA splicing, mRNA processing, RNA binding, nuclear matrix, and nucleolus (Figure 2A). Notably, these proteins showed higher abundance in both the odd and even groups compared to the RNA-depleted group, suggesting their association with NEAT1-mediated regulation of RNA splicing (Figure 2B). NEAT1 is a crucial component of paraspeckles, which are phase-separated nuclear condensates often found at the periphery of nuclear speckles [16,19]. Consistent with this, we observed the co-purification of several previously reported constituents of paraspeckles, including FUS, DDX17, NONO, and various heterogeneous nuclear ribonucleoproteins (HNRNPs), with NEAT1 (Figure 2C and Table S3) [20]. Frequently proteins that act together tend to interact physically. When we mapped the protein–protein interaction (PPI) network, we observed a concentrated cluster of RNA binding proteins involved in splicing and other regulatory processes (Figure. S3)

### Comparison of MORPH-MS with existing capture-based methods

There are various methods available for studying RNA-protein interactions, including RAP-MS [6], CHART [7], ChiRP [8], CRISPR-Assisted RNA-Protein Interaction 1Detection (CaRPID) [21], BioRBP [22], etc. These methods can be broadly categorized into three groups: ASO-based capture, aptamer-based capture, and proximity-based labelling. Aptamer-based methods such as MS2-tagged RNA affinity purification (MS2-TRAP) [23] and BioRBP [22], and proximity-based methods such as RNA-protein interaction detection (RaPID) [24], CaRPID [21], CRISPR-based RNA proximity proteomics (CBRPP) [25], and RNA proximity labelling (RPL) [26] require cloning and transfections, making them less suitable for very long RNA or difficult-to-transfect cell lines. ASO-based capturing techniques can be used for RNA of any length, making them versatile. However, they are particularly well-suited for long RNAs like NEAT1, MALAT1, and *Xist*. MORPH, along with methods like CHART [7], ChIRP [8], RAP-MS [6], HyPR-MS [9], and identification of direct RNA interacting proteins (iDRiP) [27], belongs to the ASO-based capture category. Differences between these techniques are listed in Table 1. All these methods require the use of biotinylated oligos, but they differ in way of fixation, number of oligos and method of elution (Table 1). For example, CHART [7] uses a single 20–25 nucleotide biotinylated oligo designed through RNaseH mapping and regions devoid of secondary structures whereas RAP-MS [6] utilizes SILAC-based quantitative mass spectrometry with a tiling array of non-overlapping 90 nucleotide long biotinylated probes. The longer length of individual oligos in RAP-MS increases the chance of self-complementarity and binding among the oligos, necessitating a higher hybridization temperature. ChiRP [8], iDRiP [27], and MORPH require 20–25 base pairs of complementary binding region for RNA capture. Unlike MORPH, both ChiRP [8] and iDRiP [27] use individually biotinylated 20–25 nucleotide probes, whereas iDRiP uses a reduced number of oligos compared to ChiRP. Among all the methods discussed, MORPH-MS stands out by utilizing a single biotinylated probe to capture all the complementary oligos, reducing the cost of probe synthesis by multiple folds.

**Table 1.** Comparing MORPH with different antisense oligo-based capture techniques.

Name	oligo design	Fixation	Hybridization	Elution	Remark	Citation
iDRiP-MS	Fewer number of 20–25 oligomers biotinylated DNA oligos	UV crosslinking	slowly decreasing temperature-based hybridization	Tris-Hcl at 70°C for elution	Requires a smaller number (20–25) of individually biotin labelled probes, high cost of synthesis	[26,27]
CHART	Single 20-25mers biotinylated oligo designed by RNaseH mapping	3% Formaldehyde for 30 min	urea and denhardt solution at room temperature	RNaseH elution	Requires RNAseH mapping to design a biotinylated oligo	[7]
RAP-MS	Tiling array of 90mer oligos across target RNA	UV crosslink at 254 nm	using detergents such as DDM and sodium deoxycholate at 67°C	Benzonase nuclease	longer biotinylated oligos (4–5) increases the chances of non-specific binding	[6]
ChiRP-MS	Array of 20–25 oligomers biotinylated oligo across target	3% formaldehyde for 30 min	formamide based at 37°C	excess biotin	Multiple (upto 40) biotinylated oligos increase the cost of the protocol	[8]
HyPR-MS	Biotinylated capture oligo complementary to target RNA. The oligo has a toehold sequence	1% Formaldehyde fixation	DTT buffer at 37°C for 3 h	toehold sequence mediated release	Multiple biotinylated oligos having toehold sequence, increasing the cost. Use of RNA specific toehold sequence for the release of probes	[9]
MORPH-MS	Array of 20–25 oligomers having a common sequence at 5’ end across all oligos which can be captured by a single biotin labelled Universal oligo	3% PFA for 30 min	formamide based at 37°C	Benzonase elution	Use of a universal biotinylated oligo for all the anti-sense probes. Low cost of synthesis	This study

**Figure 3.**
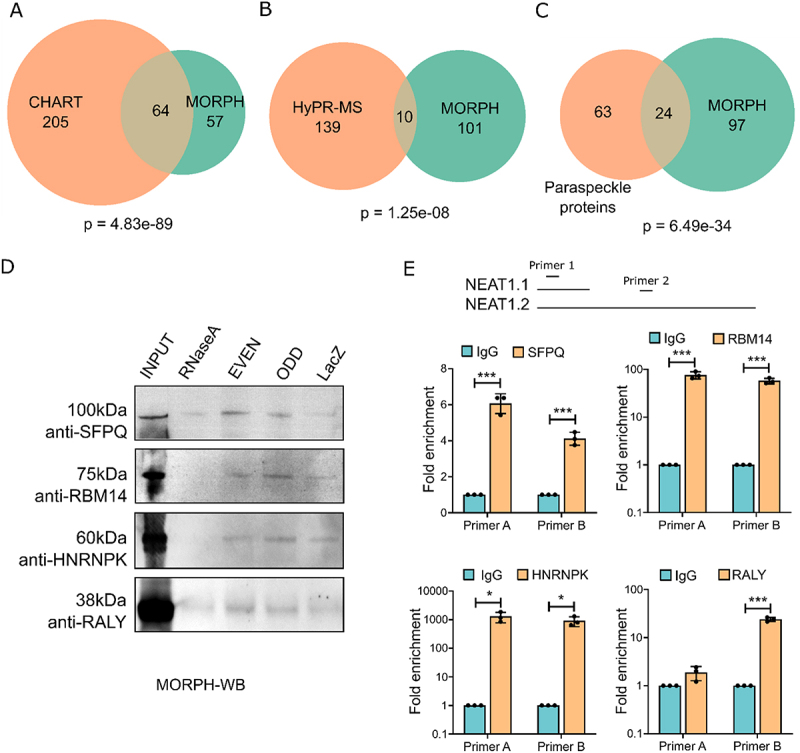
Validation of MORPH (A) venn diagram showing the number of common NEAT1 interacting proteins detected using CHART and MORPH-MS (Fisher exact test, *p* = 4.833e-89). (B) Venn diagram showing the number of common NEAT1 interacting proteins detected using CHART and HyPR-MS (Fisher exact test, *p* = 1.25e-08). (C) Venn diagram showing the number of common paraspeckle proteins detected by MORPH-MS (Fisher exact test, *p* = 6.49e-34). (D) Western blot after performing RNA pulldown via MORPH (MORPH-WB) using anti-SFPQ, anti-RBM14, anti-HNRNPK, and anti-RALY. (E) qRT-PCR using NEAT1 primers after performing RNA-IP from AC16 cell lysates using antibodies against SFPQ, RBM14, HNRNPK, and RALY. IgG was used as negative control.

MORPH-MS successfully identified 121 high-confidence NEAT1 interactors (Table S2). To validate the reliability of MORPH-MS, we compared the NEAT1 interacting proteins enriched by MORPH-MS with those identified in previous studies using other techniques, such as CHART and HyPR-MS. Remarkably, out of the 121 proteins detected by MORPH-MS, 64 were also detected by CHART (Fisher exact test, *p* = 4.83e-89) (Figure 3A). Additionally, 10 proteins were found to be common between MORPH and HyPR-MS (Fisher exact test, *p* = 1.25e-08) (Figure 3B). Among the 87 known paraspeckle proteins, MORPH successfully detected 24 proteins (Fisher exact test, *p* = 6.49e-34) (Figure 3C, Table S3). We assessed the presence of the RNA binding protein SFPQ, RMB14 and HnRNPK, known interactors of NEAT1, using western blot analysis after MORPH. Remarkably, all were enriched in the even and odd sets of probes (Figure 3D).

To validate the protein-RNA interactions, we selected a subset of interacting proteins and performed RNA immunoprecipitation (RNA-IP). The core paraspeckle components, SFPQ, RBM14, and HNRNPK, showed significantly higher enrichment of NEAT1, confirming their interaction (Figure 3E) with NEAT1.

### Novel interactors identified by MORPH-MS

**Figure 4.**
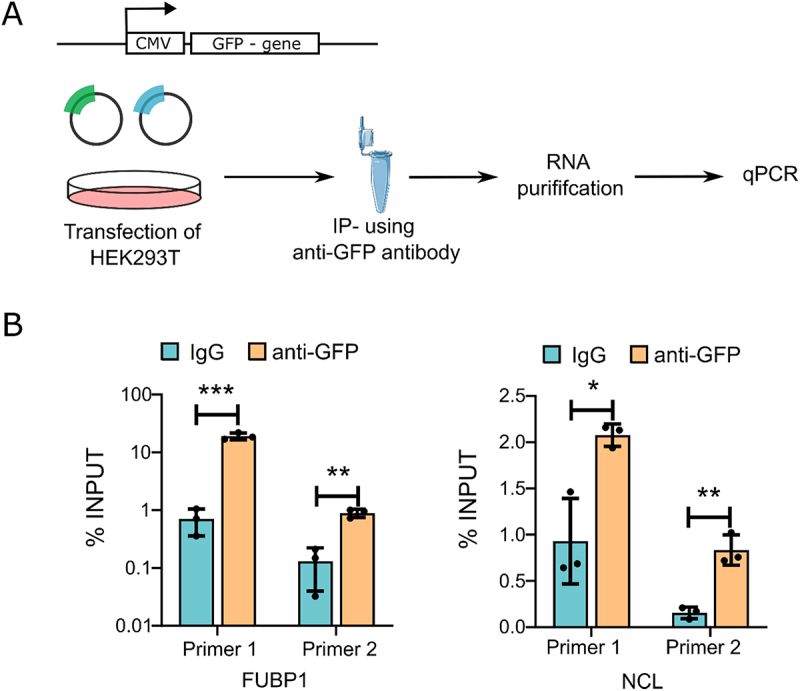
Validation of proteins by RNA-IP qPCR (A) schema depicting the RNA-IP experiment. (B) qRT-PCR using NEAT1 primers after performing RNA-IP from HEK293T cell lysates using anti-GFP antibody to enrich FUBP1 and NCL. IgG was used as the negative control.

Within the list of known NEAT1 interactors, we discovered certain proteins that had not been previously linked to either NEAT1 or paraspeckles. Two of these proteins, Far upstream element-binding protein 1 (FUBP1) and Nucleolin (NCL), were selected for further investigation. FUBP1 is traditionally recognized for its role in Myc transcription regulation [28]. However, a recent report has shown that FUBP1 also functions as a general splicing factor, strengthening its potential as a NEAT1 interacting protein [29]. Similarly, Paraspeckles were initially identified in a search for nucleolar proteins [30]. In our MORPH-MS, we consistently found NCL in both replicates. Consequently, we conducted RNA immunoprecipitation (RNA-IP) experiments for both FUBP1 and NCL, which revealed the enrichment of NEAT1 with both proteins (Figure 4). Notably, FUBP1 appeared to be a more robust NEAT1 interactor compared to NCL.

### Limitations of MORPH-MS

We employed MORPH-MS to identify RNA-protein interactions. However, by adjusting the crosslinking conditions, MORPH can be adapted to map RNA-DNA or RNA-RNA interactions as well. Since MOPRH uses formaldehyde as fixation agent, it allowed us to map both direct and indirect interators of NEAT1. However, if the objective is to specifically identify direct interactors, UV fixation can be employed instead of formaldehyde fixation. Similarly, Gluteradehyde fixation can map RNA-DNA interactions. Furthermore, when cells are cultured in labelled media, MORPH can be combined with quantitative proteomics techniques Such as stable isotope labelling of amino acids (SILAC) and isobaric labels. This allows capturing RNA from various experimental conditions from a single pulldown, similar to the strategy used in RAP-MS [6]. In this study, we employed used the whole cell extract to map NEAT1 interacting proteins. However, one can opt for nuclear or cytoplasmic extracts based on the sub-cellular location of the lncRNA of interest.

It is worth noting that MOPRH cannot be used for small RNAs since the probe size used in MOPRH itself is larger than 22nts, the method in its present form would not be able to capture small RNAs. Nevertheless, MORPH can be used for RNA of any length and abundance although, it is extremely useful for very large and abundant lncRNA because it is independent of cloning and transfections. Just like any other high throughput technique, MORPH gives a list of putative interactors. Further experiments such as RNA-IP and immunoblots are required to validate the interactions.

## Supplementary Material

Supplemental Material

## Data Availability

Processed mass spectrometry data are included as supporting files. Raw data were generated at the CSIR-Centre for Cellular and Molecular Biology. Raw data supporting the findings of this study are available from the corresponding author RK on request.
