# Supplementary material for: Multiple Oligo assisted RNA Pulldown via Hybridization followed by Mass Spectrometry (MORPH-MS) for exploring the RNA-Protein interactions
Source: RNA Biol. 2023 Dec 17;21(1):56–64. doi: 10.1080/15476286.2023.2287302 (PMC10730167; doi:10.1080/15476286.2023.2287302)
Supplement: Supplemental Material [file KRNB_A_2287302_SM7579.zip › Supplemetary figure and table legends-MORPH (1).docx]

**Supplementary figure and table legends**

**Supplementary Figure S1 (A)** Position of the anti-sense oligos, used in this study, on the *NEAT1* RNA (*NEAT1.2* isoform, 23kb) **(B)** Position of the anti-sense oligos, used in this study, on the *LacZ* RNA.

**Supplementary Figure S2** Oligos resolved in 6% PAGE gel before and after annealing. 50bp DNA ladder is loaded to confirm shift in band and the relative size of the oligo. The gel is stained with EtBr for visualizing DNA

**Supplementary Figure S3** Protein-protein interaction network map of the NEAT1 interactors

**Supplementary Figure S4** qRT-PCR using NEAT1 primers after performing RNA-IP from HEK293T cell lysates using antibodies against SFPQ, RBM14, HNRNPK and RALY. IgG are used as negative control

**Supplementary Figure S5** Silver staining of the lysates enriched after pull down using LacZ, odd, even probes with RNaseA as negative control

**Table S1.** List of the ASO targeting NEAT1 and LacZ with the common binding sequence of universal oligo

**Table S2.** List of *NEAT1* interactors detected in both replicates along with the abundance, abundance ratio of Odd/RNaseA, Even/RNaseA and the log2abundance ratio. Avg SC column indicates the spectral count observed in the CRAPome repository

**Table S3.** List of *NEAT1* interactors detected in both replicates along with the abundance, abundance ratio of Odd/LacZ, Even/LacZ and the log2abundance ratio. Proteins indicated in blue are the NEAT1 interacting partners that are commonly identified when RNaseA or LacZ were used as control. Avg SC column indicates the spectral count observed in the CRAPome repository

**Table S4.** List of paraspeckle proteins detected after MORPH-MS

**Table S5.** List of human *NEAT1* primers used for qPCR and list of cloning primers used to amplify FUBP1 and NCL.


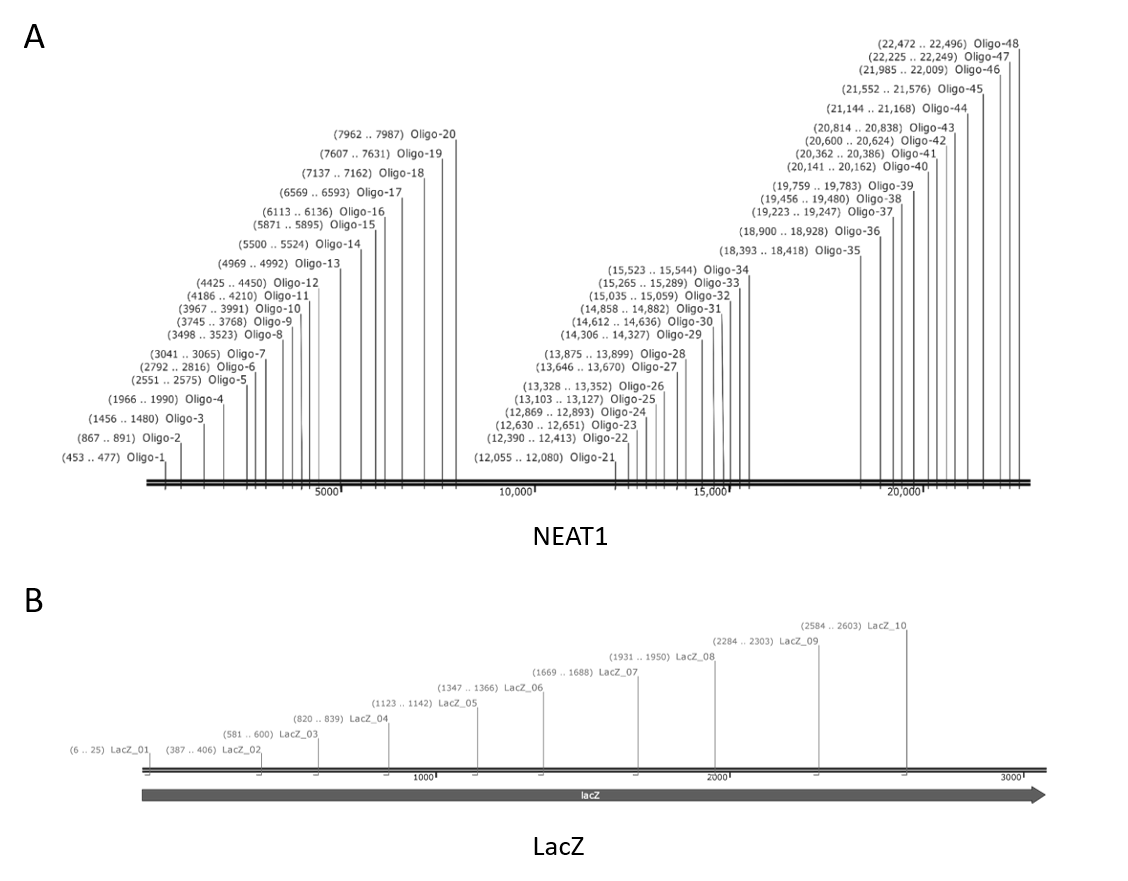


**Supplementary Figure S1. (A)** Position of the anti-sense oligos, used in this study, on the *NEAT1* RNA (*NEAT1.2* isoform, 23kb) **(B)** Position of the anti-sense oligos, used in this study, on the *LacZ* RNA.

**
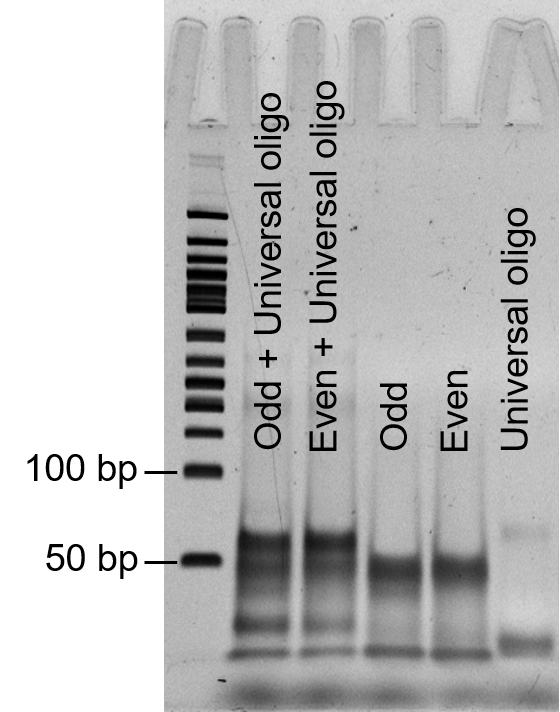
**

**Supplementary Figure S2** Oligos resolved in 6% PAGE gel before and after annealing. 50bp DNA ladder is loaded to confirm shift in band and the relative size of the oligo.

**
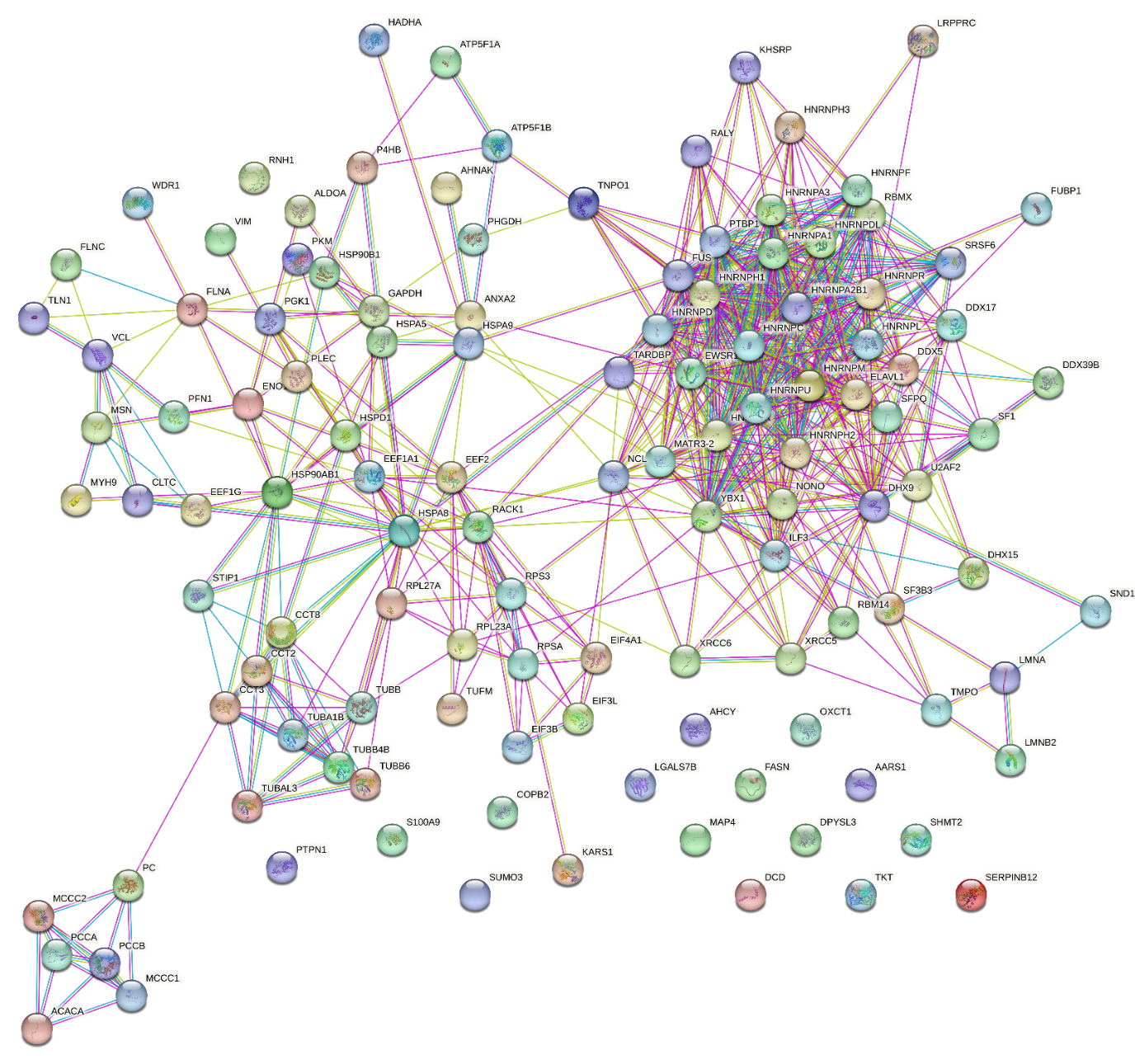
**

**Supplementary Figure S3** Protein-protein interaction network map of the NEAT1 interactors


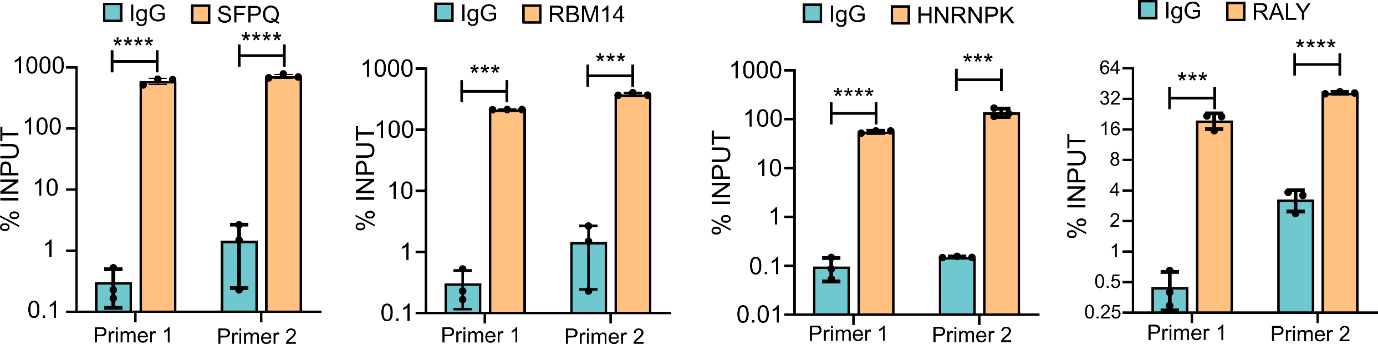


**Supplementary Figure S4** qRT-PCR using NEAT1 specific primers after RNA-IP from HEK293T cell lysates using antibodies against SFPQ, RBM14, HNRNPK and RALY. IgG are used as negative control


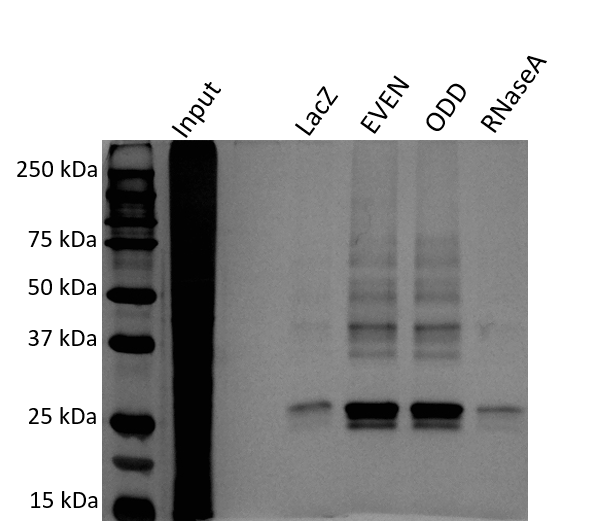


**Supplementary Figure S5** Silver staining of the lysates enriched after pull down using LacZ, odd, even probes with RNaseA as negative control.
