# Supplementary material for: Multiple Oligo assisted RNA Pulldown via Hybridization followed by Mass Spectrometry (MORPH-MS) for exploring the RNA-Protein interactions
Source: RNA Biol. 2023 Dec 17;21(1):56–64. doi: 10.1080/15476286.2023.2287302 (PMC10730167; doi:10.1080/15476286.2023.2287302)
Supplement: Supplemental Material [file KRNB_A_2287302_SM7579.zip › Table S1.pdf]

Oligos used for MORPH

**Universal oligo**	5'TEG-BIOTIN-AATGCATGTCGACGAGGTCCGAGTGTAA-TEG-BIOTIN-3'
**Neat1 targetting ASO**	
Oligo-1	ggcatggacaagttgaagattagccTTACACTCGGACCTCGTCGACATGCATT
Oligo-2	gtgagctcacaagaagagtttagcgTTACACTCGGACCTCGTCGACATGCATT
Oligo-3	gggctctggaacaagcatttattcaTTACACTCGGACCTCGTCGACATGCATT
Oligo-4	gcaagaacaaaagagcactaccggtTTACACTCGGACCTCGTCGACATGCATT
Oligo-5	accaggaggaagctggtaaagacatTTACACTCGGACCTCGTCGACATGCATT
Oligo-6	ggtcccttaaatacgttaaaagcccTTACACTCGGACCTCGTCGACATGCATT
Oligo-7	gtgaccacaaaaggttatttggttgTTACACTCGGACCTCGTCGACATGCATT
Oligo-8	cgacagtaattgtttgcatcatccccTTACACTCGGACCTCGTCGACATGCATT
Oligo-9	ggggtcgagaaatgtaacatagcaTTACACTCGGACCTCGTCGACATGCATT
Oligo-10	ggcttgagtggtttcatcacatttcTTACACTCGGACCTCGTCGACATGCATT
Oligo-11	ttctttagtgaggtaagaattacgcTTACACTCGGACCTCGTCGACATGCATT
Oligo-12	gcccaaagtttgtctcctgtaagcaaTTACACTCGGACCTCGTCGACATGCATT
Oligo-13	caggacgagaaacaaggagttctgTTACACTCGGACCTCGTCGACATGCATT
Oligo-14	ctgggcatactgaagagcctcaaccTTACACTCGGACCTCGTCGACATGCATT
Oligo-15	agccacctgccttggaacatgtaccTTACACTCGGACCTCGTCGACATGCATT
Oligo-16	aagcaaatgtgcccataacaattgTTACACTCGGACCTCGTCGACATGCATT
Oligo-17	cggaggagcaaaggttttcaatgtgTTACACTCGGACCTCGTCGACATGCATT
Oligo-18	ccctcaaggtcttctgaattgaacccTTACACTCGGACCTCGTCGACATGCATT
Oligo-19	caaatgtgtttgtgaactctgccggTTACACTCGGACCTCGTCGACATGCATT
Oligo-20	ctggagtcatttgagtctgtgaagagTTACACTCGGACCTCGTCGACATGCATT
Oligo-21	gtaggtcactgcatacatgatagatcTTACACTCGGACCTCGTCGACATGCATT
Oligo-22	ttcatacactccacagccccatccTTACACTCGGACCTCGTCGACATGCATT
Oligo-23	atatgacatgttatagagccatTTACACTCGGACCTCGTCGACATGCATT
Oligo-24	agcacgtgctttcaacccatctcacTTACACTCGGACCTCGTCGACATGCATT
Oligo-25	acaaatgtcacagacgctttcctagTTACACTCGGACCTCGTCGACATGCATT
Oligo-26	tgagtgaccctatgaggtaagagccTTACACTCGGACCTCGTCGACATGCATT
Oligo-27	catgtgcaggtatggctttgaacagTTACACTCGGACCTCGTCGACATGCATT
Oligo-28	ggcagacatgaatacagatatgcggTTACACTCGGACCTCGTCGACATGCATT
Oligo-29	tttgaaaagggttaatttccttTTACACTCGGACCTCGTCGACATGCATT
Oligo-30	aaggacaacaggctaactaacccygTTACACTCGGACCTCGTCGACATGCATT
Oligo-31	acgtgagtggatggaaagacgtaccTTACACTCGGACCTCGTCGACATGCATT
Oligo-32	gtcggcgtgacagtatcaagtaaccTTACACTCGGACCTCGTCGACATGCATT
Oligo-33	GtgctggacactagaacaggactccTTACACTCGGACCTCGTCGACATGCATT
Oligo-34	accatcataaatggaaaaccggTTACACTCGGACCTCGTCGACATGCATT
Oligo-35	gatatttcccatcataaagggacaggTTACACTCGGACCTCGTCGACATGCATT
Oligo-36	gcagaactgcttactttatctaaagctgcTTACACTCGGACCTCGTCGACATGCATT
Oligo-37	GCccacacgaaaccttacatcttccTTACACTCGGACCTCGTCGACATGCATT
Oligo-38	gatttgtgcattgccacctgaggccTTACACTCGGACCTCGTCGACATGCATT
Oligo-39	atacagatggaaggccttcatgtgcTTACACTCGGACCTCGTCGACATGCATT
Oligo-40	ccattctaactagggtcaaattTTACACTCGGACCTCGTCGACATGCATT
Oligo-41	CCaggagtgacggtgagaatgcaacTTACACTCGGACCTCGTCGACATGCATT
Oligo-42	CAgcagttgaaaataaccctcatagTTACACTCGGACCTCGTCGACATGCATT
Oligo-43	tcgtaaccacaaaatgctggtgccgTTACACTCGGACCTCGTCGACATGCATT
Oligo-44	GGgcagtcaaaacacactctgtaagTTACACTCGGACCTCGTCGACATGCATT

Oligo-45	ctgatgaatctgagcagaatcagccTTACACTCGGACCTCGTCGACATGCATT
Oligo-46	tcgaaatgaagtcggaagcacagggTTACACTCGGACCTCGTCGACATGCATT
Oligo-47	ggcttgaatttttgactctatgcagTTACACTCGGACCTCGTCGACATGCATT
Oligo-48	aagtcagaccagcaaactgaacacgTTACACTCGGACCTCGTCGACATGCATT
**LacZ targetting ASO**	
LacZ_01	ccagtgaatccgtaatcatgTTACACTCGGACCTCGTCGACATGCATT
LacZ_02	gtagccagctttcatcaacaTTACACTCGGACCTCGTCGACATGCATT
LacZ_03	atcttccagataactgccgtTTACACTCGGACCTCGTCGACATGCATT
LacZ_04	ataatttcaccgccgaaaggTTACACTCGGACCTCGTCGACATGCATT
LacZ_05	ttcatcagcaggatatcctgTTACACTCGGACCTCGTCGACATGCATT
LacZ_06	tgatcacactcgggtgattaTTACACTCGGACCTCGTCGACATGCATT
LacZ_07	aaacggggatactgacgaaaTTACACTCGGACCTCGTCGACATGCATT
LacZ_08	gttatcgctatgacggaacaTTACACTCGGACCTCGTCGACATGCATT
LacZ_09	tgtgaaagaaagcctgactgTTACACTCGGACCTCGTCGACATGCATT
LacZ_10	gtaatcgccatttgaccactTTACACTCGGACCTCGTCGACATGCATT
