# Supplementary material for: Multiple Oligo assisted RNA Pulldown via Hybridization followed by Mass Spectrometry (MORPH-MS) for exploring the RNA-Protein interactions
Source: RNA Biol. 2023 Dec 17;21(1):56–64. doi: 10.1080/15476286.2023.2287302 (PMC10730167; doi:10.1080/15476286.2023.2287302)
Supplement: Supplemental Material [file KRNB_A_2287302_SM7579.zip › Table S2.pdf]

**121 interactors (normalized with RNaseA)**																		
				Replicate 1							Replicate 2							
Uniprot Accession IDs	Entry name	# AAs	MW [kDa]	Abundances : ODD	Abundances : EVEN	Abundances : RNaseA	Abundance Ratio: ODD / RNaseA	Abundance Ratio: EVEN / RNaseA	Abundance Ratio (log2): ODD / RNaseA	Abundance Ratio (log2): EVEN / RNaseA	Abundances ODD	Abundances : EVEN	Abundances RNaseA	Abundance Ratio: ODD / RNaseA	Abundance Ratio: EVEN / RNaseA	Abundance Ratio (log2): ODD / RNaseA	Abundance Ratio (log2): EVEN / RNaseA	Avg SC
Q9Y262	EIF3L_HUMAN	564	66.7	279.3	20.7		100	100	6.64	6.64	114.6	185.4		100	100	6.64	6.64	5.3
P52597	HNRPF_HUMAN	415	45.6	250.8	49.2		100	100	6.64	6.64	152.5	147.5		100	100	6.64	6.64	9.2
P55795	HNRH2_HUMAN	449	49.2	221.5	78.5		100	100	6.64	6.64	164	136		100	100	6.64	6.64	6.8
Q15637	SF01_HUMAN	639	68.3	233.6	53.6	12.8	18.31	4.2	4.19	2.07	168.7	131.3		100	100	6.64	6.64	7.9
P26368	U2AF2_HUMAN	475	53.5	255.6	44.4		100	100	6.64	6.64	178.6	121.4		100	100	6.64	6.64	4.5
Q9UKM9	RALY_HUMAN	306	32.4	234.6	65.4		100	100	6.64	6.64	142.1	148.4	9.5	11.274	11.392	3.49	3.51	3.6
Q13247	SRSF6_HUMAN	344	39.6	241.2	38.7	20.1	20.853	3.361	4.38	1.75	182.9	114.1	3	15.29	11.149	3.93	3.48	3.9
P31942	HNRH3_HUMAN	346	36.9	198.6	76.2	25.2	9.497	4.639	3.25	2.21	155	129.6	15.4	10.33	8.409	3.37	3.07	8
P31943	HNRH1_HUMAN	449	49.2	218.2	75.8	6	32.127	11.36	5.01	3.51	154.9	132.9	12.2	8.511	8.213	3.09	3.04	13.5
Q13148	TADBP_HUMAN	414	44.7	215.6	79.1	5.3	40.604	14.906	5.34	3.9	116.3	157.9	25.8	4.507	6.119	2.17	2.61	3.4
P07910	HNRPC_HUMAN	306	33.7	210.1	78	11.8	40.689	15.035	5.35	3.91	156	120.2	23.8	7.365	5.965	2.88	2.58	9.3
Q01844	EWS_HUMAN	656	68.4	269.7	30.3		100	100	6.64	6.64	163.7	123.4	12.9	7.683	5.829	2.94	2.54	17.1
P17844	DDX5_HUMAN	614	69.1	273.6	23.7	2.6	29.67	8.739	4.89	3.13	128.3	139.1	32.5	4.723	5.649	2.24	2.5	11.1
P38159	RBMX_HUMAN	391	42.3	238.5	60.4	1.1	61.954	10.074	5.95	3.33	160.2	125.1	14.7	6.101	5.365	2.61	2.42	7.5
O14979	HNRDL_HUMAN	420	46.4	247.2	49.1	3.7	24.404	8.824	4.61	3.14	149.2	127.3	23.5	6.081	5.37	2.6	2.42	4.6
P22626	ROA2_HUMAN	353	37.4	193.6	94.9	11.5	18.441	5.333	4.2	2.41	153.2	113.4	33.4	7.587	5.252	2.92	2.39	16.8
P51991	ROA3_HUMAN	378	39.6	199.5	88.6	11.9	50.1	9.888	5.65	3.31	162.2	108.5	29.3	6.784	4.907	2.76	2.29	6.6
Q15717	ELAV1_HUMAN	326	36.1	193	98.6	8.4	23.071	11.788	4.53	3.56	162.9	117.3	19.8	5.602	4.822	2.49	2.27	4.9
Q96PK6	RBM14_HUMAN	669	69.4	225.7	67.1	7.2	45.047	10.737	5.49	3.42	139.9	137.5	22.6	4.787	4.583	2.26	2.2	9.7
Q92945	FUBP2_HUMAN	711	73.1	237.1	56.4	6.5	28.988	7.64	4.86	2.93	143.3	119.6	37	4.066	4.589	2.02	2.2	11.4
Q15233	NONO_HUMAN	471	54.2	206.3	88	5.7	40.309	15.797	5.33	3.98	147.8	134.9	17.4	5.796	4.575	2.54	2.19	22.4
P40939	ECHA_HUMAN	763	82.9	244.6	39.3	16.1	15.234	2.449	3.93	1.29	105.2	159.1	35.8	2.941	4.45	1.56	2.15	7.4
Q96AE4	FUBP1_HUMAN	644	67.5	220.9	72	7.1	27.415	9.708	4.78	3.28	147.4	122.7	29.9	5.088	4.411	2.35	2.14	4.6
P61978	HNRPK_HUMAN	463	50.9	216.5	70.4	13.1	14.254	4.294	3.83	2.1	145.9	110.6	43.4	5.41	4.331	2.44	2.11	23.2
P35637	FUS_HUMAN	526	53.4	212.3	76.7	11	9.245	3.954	3.21	1.98	132.4	132.1	35.5	5.41	4.297	2.44	2.1	19
P09651	ROA1_HUMAN	372	38.7	157.9	121.7	20.3	7.632	8.781	2.93	3.13	173.2	99.3	27.5	6.758	4.08	2.76	2.03	12.8
P23526	SAHH_HUMAN	432	47.7	246.5	33.6	19.9	12.381	1.686	3.63	0.75	166.1	112.2	21.7	5.423	4.025	2.44	2.01	9.3
Q92841	DDX17_HUMAN	729	80.2	220	50.1	30	6.114	2.676	2.61	1.42	127.7	126.3	45.9	4.022	3.884	2.01	1.96	10.8
P52272	HNRPM_HUMAN	730	77.5	224.7	71.7	3.6	53.317	13.837	5.74	3.79	120.2	143.5	36.4	3.217	3.625	1.69	1.86	17.6
P14866	HNRPL_HUMAN	589	64.1	241.8	52.9	5.3	43.738	6.637	5.45	2.73	139.3	130.9	29.8	4.554	3.427	2.19	1.78	12.4
P43243	MATR3_HUMAN	847	94.6	229.7	60.1	10.3	20.527	8.513	4.36	3.09	161.8	87.3	51	3.848	3.387	1.94	1.76	10.4
P26599	PTBP1_HUMAN	531	57.2	238.3	53.7	8	39.77	8.61	5.31	3.11	130.4	128.8	40.8	4.533	3.251	2.18	1.7	10.2
Q00839	HNRPU_HUMAN	825	90.5	226.8	57.2	15.9	28.306	6.881	4.82	2.78	153	104.3	42.7	4.006	3.214	2	1.68	18.8
Q14103	HNRPD_HUMAN	355	38.4	243.8	50.5	5.7	42.891	8.883	5.42	3.15	137.3	123.1	39.7	3.458	3.1	1.79	1.63	6.3
P18031	PTN1_HUMAN	435	49.9	221.6	59.2	19.2	11.511	3.076	3.52	1.62	96.9	150.8	52.3	1.853	2.884	0.89	1.53	2.4
Q9HCC0	MCCB_HUMAN	563	61.3	168.2	70.3	61.5	2.467	1.22	1.3	0.29	108.3	147.5	44.1	1.807	2.801	0.85	1.49	77
Q13838	DX39B_HUMAN	428	49	264.1	30.2	5.6	29.93	5.343	4.9	2.42	165.5	96.5	38	4.226	2.794	2.08	1.48	8.4
P34897	GLYM_HUMAN	504	56	234.8	51.3	13.9	17.365	3.709	4.12	1.89	130.6	128.4	41	3.282	2.761	1.71	1.47	9.7

P46776	RL27A_HUMAN	148	16.6	226.2	50.8	23	9.841	2.212	3.3	1.15	199.2	69.5	31.3	6.258	2.606	2.65	1.38	3.5
P05166	PCCB_HUMAN	539	58.2	184.5	65.7	49.7	3.328	1.038	1.73	0.05	104.4	116.7	78.9	2.441	2.593	1.29	1.37	75.8
P07237	PDIA1_HUMAN	508	57.1	218	54.1	27.9	7.805	1.935	2.96	0.95	86	159	55	1.488	2.471	0.57	1.31	13.1
P26641	EF1G_HUMAN	437	50.1	213.7	50.2	36.1	7.901	1.637	2.98	0.71	159.6	95.6	44.8	4.208	2.448	2.07	1.29	8.9
P78371	TCPB_HUMAN	535	57.5	273.1	23.1	3.8	45.051	3.921	5.49	1.97	121.9	120.2	57.8	2.694	2.419	1.43	1.27	19.3
O43390	HNRPR_HUMAN	633	70.9	262.1	35.7	2.2	40.239	7.938	5.33	2.99	125	124.3	50.7	2.405	2.409	1.27	1.27	9.1
P49411	EFTU_HUMAN	452	49.5	169.9	66.5	63.6	3.854	1.34	1.95	0.42	117.2	119.2	63.6	1.933	2.301	0.95	1.2	11.7
P23246	SFPQ_HUMAN	707	76.1	218.4	74.6	7	28.005	7.746	4.81	2.95	141.1	111.5	47.4	3.473	2.207	1.8	1.14	16.6
P38646	GRP75_HUMAN	679	73.6	220.3	59.2	20.5	9.286	2.751	3.21	1.46	116	104.2	79.7	2.872	2.188	1.52	1.13	26.2
P25705	ATPA_HUMAN	553	59.7	242.3	42.5	15.2	15.248	3.404	3.93	1.77	132.9	115.3	51.8	2.47	2.17	1.3	1.12	15.9
P50990	TCPQ_HUMAN	548	59.6	241.6	41.3	17.1	14.886	2.434	3.9	1.28	138.5	116.6	44.9	2.35	2.176	1.23	1.12	36.7
P10809	CH60_HUMAN	573	61	238.7	44.2	17.1	13.548	2.643	3.76	1.4	121.6	111.7	66.7	1.965	2.166	0.97	1.12	22.2
P23396	RS3_HUMAN	243	26.7	241.9	44.3	13.7	13.947	3.158	3.8	1.66	165	92.4	42.6	3.829	2.164	1.94	1.11	15.7
P07737	PROF1_HUMAN	140	15	239.6	45.1	15.3	15.694	2.953	3.97	1.56	179	80.5	40.5	4.912	2.135	2.3	1.09	6.6
P06733	ENOA_HUMAN	434	47.1	224.5	54.1	21.4	6.77	1.8	2.76	0.85	138.9	98.4	62.7	2.83	2.076	1.5	1.05	19.8
Q92973	TNPO1_HUMAN	898	102.3	211.3	62.5	26.2	8.057	2.382	3.01	1.25	146.9	103.8	49.4	2.976	2.052	1.57	1.04	2.3
O75083	WDR1_HUMAN	606	66.2	247.3	37.7	15.1	14.911	2.445	3.9	1.29	141.1	115.2	43.7	2.463	2.06	1.3	1.04	6.1
P63244	RACK1_HUMAN	317	35.1	258.1	30.3	11.5	22.391	2.63	4.48	1.4	178.2	86	35.7	3.586	2.045	1.84	1.03	6.8
P62750	RL23A_HUMAN	156	17.7	237.3	47.1	15.6	15.197	3.016	3.93	1.59	170.3	86.2	43.4	3.993	2.022	2	1.02	6.4
P08865	RSSA_HUMAN	295	32.8	158.5	132.4	9.1	3.088	1.271	1.63	0.35	161.9	93.3	44.8	2.93	2.016	1.55	1.01	7.3
Q03252	LMNB2_HUMAN	620	69.9	185	88	27	6.848	3.256	2.78	1.7	109.8	129.1	61.1	1.8	1.994	0.85	1	5
P60842	IF4A1_HUMAN	406	46.1	241.8	34.5	23.6	12.007	1.466	3.59	0.55	152	97.6	50.4	2.542	1.978	1.35	0.98	12.6
O43175	SERA_HUMAN	533	56.6	244.4	45	10.7	22.864	4.206	4.52	2.07	131	138.6	30.4	1.729	1.959	0.79	0.97	6.2
Q12906	ILF3_HUMAN	894	95.3	245.4	46.5	8.1	36.461	8.107	5.19	3.02	151.1	98	51	2.779	1.948	1.47	0.96	10.1
P00558	PGK1_HUMAN	417	44.6	247.5	40.1	12.4	15.65	3.367	3.97	1.75	151	96	53	3.061	1.922	1.61	0.94	9
Q9BUF5	TBB6_HUMAN	446	49.8	186.8	55.5	57.7	6.521	1.622	2.71	0.7	127.1	81.4	91.6	2.004	1.923	1	0.94	15.3
Q13085	ACACA_HUMAN	2346	265.4	188.4	65.8	45.8	5.411	1.746	2.44	0.8	108.4	116.1	75.6	1.657	1.908	0.73	0.93	315.2
P06576	ATPB_HUMAN	529	56.5	238.5	50.7	10.8	13.73	2.873	3.78	1.52	152.4	96.6	51	3.71	1.844	1.89	0.88	14.7
P68104	EF1A1_HUMAN	462	50.1	242.2	39.6	18.2	15.863	3.894	3.99	1.96	147.9	83.5	68.6	2.729	1.749	1.45	0.81	30
P13010	XRCC5_HUMAN	732	82.7	247.6	40.5	11.9	20.887	3.419	4.38	1.77	124.8	102.8	72.5	2.069	1.723	1.05	0.79	9.1
P13489	RINI_HUMAN	461	49.9	251	41.5	7.5	20.886	3.231	4.38	1.69	165	85.2	49.9	3.247	1.686	1.7	0.75	9.8
P35606	COPB2_HUMAN	906	102.4	230.7	55.4	13.9	16.624	3.994	4.06	2	139.1	101.5	59.5	2.256	1.686	1.17	0.75	3.6
P55884	EIF3B_HUMAN	814	92.4	258.9	28.9	12.3	21.111	2.355	4.4	1.24	133.3	107.3	59.4	2.185	1.665	1.13	0.74	5
P26038	MOES_HUMAN	577	67.8	199.9	53.7	46.5	4.301	1.155	2.1	0.21	129	106.7	64.3	2.187	1.668	1.13	0.74	4.4
P14618	KPYM_HUMAN	531	57.9	250.8	35.9	13.3	23.336	3.426	4.54	1.78	137.3	79.5	83.2	2.25	1.622	1.17	0.7	19.6
P49368	TCPG_HUMAN	545	60.5	229.6	44	26.4	11.135	2.134	3.48	1.09	134.2	105.1	60.7	2.54	1.618	1.34	0.69	15
P67809	YBOX1_HUMAN	324	35.9	190.7	66	43.3	4.018	1.544	2.01	0.63	144.6	94	61.4	2.771	1.557	1.47	0.64	8.6
Q7KZF4	SND1_HUMAN	910	101.9	259.6	40.4		100	100	6.64	6.64	143.1	95.8	61.1	2.295	1.537	1.2	0.62	8.4
Q15046	SYK_HUMAN	597	68	258.5	32.6	8.9	29.123	3.67	4.86	1.88	111.9	113.3	74.8	1.484	1.507	0.57	0.59	6.9
P29401	TKT_HUMAN	623	67.8	268	27.1	4.9	25.845	3.025	4.69	1.6	125.5	109.5	65	1.086	1.5	0.12	0.59	7.5
P12956	XRCC6_HUMAN	609	69.8	276.9	19.3	3.8	45.586	4.303	5.51	2.11	125.7	101.7	72.6	1.858	1.496	0.89	0.58	11.7
Q96RQ3	MCCA_HUMAN	725	80.4	208.7	57.8	33.4	10.521	2.591	3.4	1.37	99.8	111.6	88.6	1.322	1.488	0.4	0.57	175.8
A6NHL2	TBAL3_HUMAN	446	49.9	115.6	133	51.4	2.246	2.585	1.17	1.37	170.9	76.8	52.3	3.268	1.469	1.71	0.56	4.6
P11498	PYC_HUMAN	1178	129.6	160.2	73.5	66.3	3.224	1.402	1.69	0.49	123.8	89.9	86.2	1.581	1.471	0.66	0.56	290.8
P05165	PCCA_HUMAN	728	80	171.4	75.8	52.8	3.822	1.624	1.93	0.7	99.4	108.9	91.8	1.303	1.466	0.38	0.55	151.8

P55809	SCOT1_HUMAN	520	56.1	237.1	47	15.8	14.997	2.975	3.91	1.57	123.6	104.5	71.9	1.719	1.453	0.78	0.54	5.6
P42166	LAP2A_HUMAN	694	75.4	229.1	61.2	9.7	23.577	6.302	4.56	2.66	131.8	99	69.2	2.089	1.446	1.06	0.53	17.7
P04406	G3P_HUMAN	335	36	237.4	44.2	18.4	11.716	2.489	3.55	1.32	138.6	95.9	65.6	2.032	1.429	1.02	0.52	14.2
P06702	S10A9_HUMAN	114	13.2	263.6	20.6	15.9	16.625	1.299	4.06	0.38	99.9	116.4	83.7	1.597	1.429	0.68	0.52	3.4
P08670	VIME_HUMAN	466	53.6	167.1	80.5	52.5	3.785	1.672	1.92	0.74	140.9	83.7	75.4	2.311	1.424	1.21	0.51	39.2
P11021	BIP_HUMAN	654	72.3	229	55.4	15.6	15.592	4.032	3.96	2.01	126.2	91.2	82.6	1.69	1.408	0.76	0.49	23.9
P31948	STIP1_HUMAN	543	62.6	264.4	31.2	4.4	60.511	7.151	5.92	2.84	132.9	95.9	71.3	1.776	1.374	0.83	0.46	8.3
P11142	HSP7C_HUMAN	646	70.9	230.3	47.2	22.5	12.653	2.186	3.66	1.13	132.3	93.5	74.2	1.695	1.362	0.76	0.45	46.9
Q15393	SF3B3_HUMAN	1217	135.5	245.3	44.7	10	24.596	4.479	4.62	2.16	142.5	90.1	67.3	2.117	1.338	1.08	0.42	13
P47929	LEG7_HUMAN	136	15.1	192.8	15.2	92	28.843	2.271	4.85	1.18	91.5	122.8	85.7	1.106	1.333	0.14	0.42	3.4
P04075	ALDOA_HUMAN	364	39.4	154.6	75.2	70.2	2.203	1.071	1.14	0.1	148	65.8	86.1	2.428	1.326	1.28	0.41	11.2
Q08211	DHX9_HUMAN	1270	140.9	220.6	54	25.4	11.561	2.424	3.53	1.28	131.7	75	93.4	2.782	1.31	1.48	0.39	21.4
P02545	LMNA_HUMAN	664	74.1	223.1	47.8	29	7.51	2.053	2.91	1.04	139.7	89.4	70.9	1.96	1.305	0.97	0.38	9
Q96P63	SPB12_HUMAN	405	46.2	278.1	21.9		100	100	6.64	6.64	94	115.4	90.6	1.108	1.297	0.15	0.37	3.3
P35579	MYH9_HUMAN	1960	226.4	219.2	54.9	25.9	16.915	3.074	4.08	1.62	134	71.5	94.5	2.28	1.265	1.19	0.34	51.8
P68363	TBA1B_HUMAN	451	50.1	226.1	50.4	23.5	9.365	2.537	3.23	1.34	148.9	89.1	61.9	1.737	1.269	0.8	0.34	47.2
P68371	TBB4B_HUMAN	445	49.8	205.6	54.4	40	8.359	1.994	3.06	1	133.1	92.9	74	1.799	1.256	0.85	0.33	40
P07355	ANXA2_HUMAN	339	38.6	180.9	55.7	63.3	5.321	1.876	2.41	0.91	166.3	73.9	59.8	2.564	1.233	1.36	0.3	9.2
Q14195	DPYL3_HUMAN	570	61.9	257.7	29.4	12.9	9.917	1.724	3.31	0.79	81.7	171.2	47.1	1.736	1.233	0.8	0.3	6.2
P13639	EF2_HUMAN	858	95.3	246.6	35.3	18.2	22.42	2.701	4.49	1.43	148.9	75.4	75.8	2.504	1.227	1.32	0.29	20.8
P49588	SYAC_HUMAN	968	106.7	254.4	35.6	10	25.32	3.54	4.66	1.82	127.2	94.6	78.2	2.278	1.221	1.19	0.29	4.5
P55854	SUMO3_HUMAN	103	11.6	227.5	43	29.5	7.706	1.456	2.95	0.54	151.5	81.5	67	2.26	1.217	1.18	0.28	1.4
P08238	HS90B_HUMAN	724	83.2	218.7	48.5	32.9	19.293	2.662	4.27	1.41	137.2	84	78.9	1.843	1.209	0.88	0.27	34.8
O43143	DHX15_HUMAN	795	90.9	260.5	34.5	5	52.051	6.886	5.7	2.78	150.1	75.7	74.2	1.773	1.173	0.83	0.23	11
Q00610	CLH1_HUMAN	1675	191.5	239.8	43.9	16.4	23.912	3.848	4.58	1.94	119.1	68.9	112	1.68	1.17	0.75	0.23	18.6
P42704	LPPRC_HUMAN	1394	157.8	254.6	45.4		100	100	6.64	6.64	149.5	88.7	61.8	2.616	1.108	1.39	0.15	11.3
P49327	FAS_HUMAN	2511	273.3	243.5	43.1	13.4	20.021	2.799	4.32	1.48	130.7	61.7	107.7	2.206	1.108	1.14	0.15	91.3
Q14315	FLNC_HUMAN	2725	290.8	244.2	44	11.8	17.384	2.994	4.12	1.58	138.1	81.9	80	1.93	1.092	0.95	0.13	14.7
P18206	VINC_HUMAN	1134	123.7	238.9	48.1	13	17.649	3.763	4.14	1.91	147.2	80.7	72.1	2.11	1.075	1.08	0.1	4.1
P19338	NUCL_HUMAN	710	76.6	218.1	58	23.9	8.484	2.925	3.08	1.55	146.6	80.3	73.1	2.098	1.068	1.07	0.09	25.4
Q9Y490	TLN1_HUMAN	2541	269.6	250.3	40.9	8.8	24.78	4.718	4.63	2.24	148	59.4	92.6	2.076	1.065	1.05	0.09	24.6
P27816	MAP4_HUMAN	1152	120.9	249.1	33.6	17.3	14.419	1.945	3.85	0.96	149.2	82.3	68.5	2.01	1.066	1.01	0.09	22
P07437	TBB5_HUMAN	444	49.6	221.9	49.1	29	5.578	1.386	2.48	0.47	152.3	77.8	69.8	1.981	1.065	0.99	0.09	47.2
Q15149	PLEC_HUMAN	4684	531.5	217.4	82.6		100	100	6.64	6.64	139.6	72.2	88.1	1.938	1.064	0.95	0.09	22
P21333	FLNA_HUMAN	2647	280.6	222.6	54.3	23.1	14.344	2.379	3.84	1.25	137.1	87.3	75.6	1.889	1.06	0.92	0.08	174.1
P81605	DCD_HUMAN	110	11.3	249	23.5	27.5	24.532	2.935	4.62	1.55	119.6	98.7	81.7	1.209	1.028	0.27	0.04	3.7
P14625	ENPL_HUMAN	803	92.4	209.8	68.5	21.7	9.99	3.278	3.32	1.71	138.9	81.8	79.3	1.759	1.022	0.81	0.03	14.4
Q09666	AHNK_HUMAN	5890	628.7	229.7	57.3	13	17.616	4.395	4.14	2.14	131.3	67.5	101.3	1.648	1.01	0.72	0.01	206
