# Supplementary material for: Multiple Oligo assisted RNA Pulldown via Hybridization followed by Mass Spectrometry (MORPH-MS) for exploring the RNA-Protein interactions
Source: RNA Biol. 2023 Dec 17;21(1):56–64. doi: 10.1080/15476286.2023.2287302 (PMC10730167; doi:10.1080/15476286.2023.2287302)
Supplement: Supplemental Material [file KRNB_A_2287302_SM7579.zip › Table S3.pdf]

**NEAT1 interactors after LacZ normalization**																		
				Replicate 1							Replicate 2							
Accession	Entry name	# AAs	MW [kDa]	Abundances (Grouped): EVEN	Abundances (Grouped): ODD	Abundances (Grouped): LacZ	Abundance Ratio: (EVEN) / (LacZ)	Abundance Ratio: (ODD) / (LacZ)	Abundance Ratio (log2): (EVEN) / (LacZ)	Abundance Ratio (log2): (ODD) / (LacZ)	Abundances (Grouped): EVEN	Abundances (Grouped): ODD	Abundances (Grouped): LacZ	Abundance Ratio: (EVEN) / (LacZ)	Abundance Ratio: (ODD) / (LacZ)	Abundance Ratio (log2): (EVEN) / (LacZ)	Abundance Ratio (log2): (ODD) / (LacZ)	Avg SC
P55795	HNRH2_HUMAN	449	49.2		163.2		100	100	6.64	6.64	78.6	221.4		100	100	6.64	6.64	6.8
A6NHL2	TBAL3_HUMAN	446	49.9	115.5	184.5		100	100	6.64	6.64	135.8	117.7	46.5	2.918	2.53	1.54	1.34	4.6
P40939	ECHA_HUMAN	763	82.9	181.3	118.7		100	100	6.64	6.64	41.6	258.4		100	100	6.64	6.64	7.4
Q01844	EWS_HUMAN	656	68.4	128.8	169.1	2	38.359	50.046	5.26	5.65	30.3	269.7		100	100	6.64	6.64	17.1
Q13148	TADBP_HUMAN	414	44.7	168.4	122.8	8.9	18.984	13.842	4.25	3.79	70.3	191	38.7	1.815	4.933	0.86	2.3	3.4
P35637	FUS_HUMAN	526	53.4	127.1	158	14.9	9.296	10.267	3.22	3.36	72.8	195.1	32.2	2.172	6.499	1.12	2.7	19
P31943	HNRH1_HUMAN	449	49.2	140.1	146.8		11.022	10.194	6.64	6.64	67	192.6	40.3	1.309	7.319	0.39	2.87	13.5
Q96AE4	FUBP1_HUMAN	644	67.5	131.2	154.4	14.5	7.621	9.515	2.93	3.25	60.6	185.5	53.9	1.012	2.726	0.02	1.45	4.6
Q15233	NONO_HUMAN	471	54.2	129.7	138.6	31.8	8.599	8.677	3.1	3.12	84.1	196.7	19.2	2.488	7.076	1.31	2.82	22.4
Q96PK6	RBM14_HUMAN	669	69.4	147.4	142.1	10.5	10.418	8.592	3.38	3.1	66.5	223.4	10.1	2.394	9.067	1.26	3.18	9.7
Q96I24	FUBP3_HUMAN	572	61.6	136.5	152.5	11	8.152	8.063	3.03	3.01	59.7	188.3	52	1.149	3.622	0.2	1.86	3
P17844	DDX5_HUMAN	614	69.1	148.7	129.8	21.4	8.432	6.746	3.08	2.75	19	218.5	62.5	1.296	4.694	0.37	2.23	11.1
P23246	SFPQ_HUMAN	707	76.1	117.7	146.6	35.7	4.825	5.872	2.27	2.55	71.3	199.4	29.3	2.897	6.95	1.53	2.8	16.6
Q92945	FUBP2_HUMAN	711	73.1	127.4	151.7	20.9	4.598	5.526	2.2	2.47	50.6	206.6	42.8	1.184	4.757	0.24	2.25	11.4
P38159	RBMX_HUMAN	391	42.3	117.2	143.2	39.7	3.698	5.008	1.89	2.32	52.3	206.1	41.6	1.054	4.566	0.08	2.19	7.5
P52272	HNRPM_HUMAN	730	77.5	147.2	117.4	35.4	7.501	4.794	2.91	2.26	64.5	200.2	35.3	1.796	6.028	0.84	2.59	17.6
P61978	HNRPK_HUMAN	463	50.9	111.4	146	42.6	3.425	4.762	1.78	2.25	61.8	191.6	46.6	1.043	3.277	0.06	1.71	23.2
P31942	HNRH3_HUMAN	346	36.9	127.3	145.6	27.1	3.982	4.684	1.99	2.23	69.8	176.3	53.9	1.548	4.829	0.63	2.27	8
P22626	ROA2_HUMAN	353	37.4	109.7	145.8	44.5	2.719	4.464	1.44	2.16	85.2	173.7	41.1	1.537	3.397	0.62	1.76	16.8
P51991	ROA3_HUMAN	378	39.6	102.8	155.5	41.7	2.301	4.088	1.2	2.18	76.5	171.8	51.7	1.159	4.262	0.21	2.09	6.6
P07910	HNRPC_HUMAN	306	33.7	116	144.9	39.1	3.303	3.999	1.72	2	75.5	202.9	21.7	1.337	4.645	0.42	2.22	9.3
P11498	PYC_HUMAN	1178	129.6	105.4	148.6	46	2.482	3.771	1.31	1.91	79.4	161.7	58.9	1.116	3.437	0.16	1.78	290.8
P09651	ROA1_HUMAN	372	38.7	93	163.2	43.8	2.512	3.695	1.33	1.89	102.6	132.8	64.6	1.366	1.831	0.45	0.87	12.8
P42704	LPPRC_HUMAN	1394	157.8	64	187.7	48.3	2.076	3.691	1.05	1.88	45.5	254.5		100	100	6.64	6.64	11.3
Q92841	DDX17_HUMAN	729	80.2	66.5	178.2	55.3	4.621	3.292	2.21	1.72	47.1	213.7	39.2	1.119	6.249	0.16	2.64	10.8
P26599	PTBP1_HUMAN	531	57.2	146	122.6	31.5	3.908	3.136	1.97	1.65	46.7	206.8	46.4	1.19	5.649	0.25	2.5	10.2
P68363	TBA1B_HUMAN	451	50.1	89.7	158.4	51.9	1.729	3.052	0.79	1.61	111.3	156.4	32.3	1.147	4.269	0.2	2.09	47.2
Q9HCC0	MCCB_HUMAN	563	61.3	161.3	115.3	23.4	4.996	2.928	2.32	1.55	73.9	186.2	39.9	1.058	4.975	0.08	2.31	77
Q13085	ACACA_HUMAN	2346	265.4	131.7	119.4	48.9	3.725	2.847	1.9	1.51	58.4	170.6	71	1.001	3.318	0	1.73	315.2
P05166	PCCB_HUMAN	539	58.2	136.7	121.5	46.7	2.846	2.84	0.19	1.51	66.6	186.7	46.7	1.056	3.743	0.08	1.9	75.8
Q15717	ELAV1_HUMAN	326	36.1	108.1	148.6	43.3	2.035	2.34	1.03	1.23	101.6	198.4		100	100	6.64	6.64	4.9
Q9UKM9	RALY_HUMAN	306	32.4	132.8	125.8	39.1	2.332	2.284	1.72	1.19	65.5	234.5		100	100	6.64	6.64	3.6
P43243	MATR3_HUMAN	847	94.6	84.4	159.6	56	2.85	1.978	1.51	0.98	55.1	210.3	34.6	1.312	5.103	0.39	2.35	10.4
