# Supplementary material for: Multiple Oligo assisted RNA Pulldown via Hybridization followed by Mass Spectrometry (MORPH-MS) for exploring the RNA-Protein interactions
Source: RNA Biol. 2023 Dec 17;21(1):56–64. doi: 10.1080/15476286.2023.2287302 (PMC10730167; doi:10.1080/15476286.2023.2287302)
Supplement: Supplemental Material [file KRNB_A_2287302_SM7579.zip › Table S4.pdf]

PSP proteins detected by MORPH

Uniprot acession ID	gene name
Q92841	DDX17
Q15717	ELAVL1
Q01844	EWSR1
P35637	FUS
P09651	HNRNPA1
P22626	HNRNPA2B1
P51991	HNRNPA3
P07910	HNRNPC
O14979	HNRNPDL
P52597	HNRNPF
P31943	HNRNPH1
P31942	HNRNPH3
P61978	HNRNPK
O43390	HNRNPR
Q00839	HNRNPU
P43243	MATR3
Q15233	NONO
P26599	PTBP1
Q96PK6	RBM14
P38159	RBMX
P23246	SFPQ
Q13148	TDP43
P67809	YBX1
Q13148	TARDBP
