# Supplementary material for: Multiple Oligo assisted RNA Pulldown via Hybridization followed by Mass Spectrometry (MORPH-MS) for exploring the RNA-Protein interactions
Source: RNA Biol. 2023 Dec 17;21(1):56–64. doi: 10.1080/15476286.2023.2287302 (PMC10730167; doi:10.1080/15476286.2023.2287302)
Supplement: Supplemental Material [file KRNB_A_2287302_SM7579.zip › Table S5.pdf]

Primers used for Neat1 detection using qPCR

HU_Neat1_1 Primer-A Fwd	5’-CTTCCTCCCTTTAACTTATCCATTCAC-3’
HU_Neat1_1 Primer-A rev	5’-CTCTTCCTCCACCATTACCAACAATAC-3’
HU_Neat1_2 Primer-B Fwd	5’-CAGTTAGTTTATCAGTTCTCCCATCCA-3’
HU_Neat1_2 Primer-B rev	5’-GTTGTTGTCGTC-ACCTTTCAACTCT-3’

Primers used for cloning NCL and FUBP1

HU-NCL-XhoI	AATGGCATCTCGAGGTATGGTGAAGCTCGCGAAGGCAGG
HU-NCL-KpnI	TGGACCGGGTACCCTATTCAAACTTCGTCTTCTTTCC
FUBP1-HindIII	GGTAAATCGAAGCTTCGATGGCAGACTATTCAACAGTGCC
FUBP1-SalI	ATTCGATTGTCGACTTATTGGCCCTGAGGTGCTGGAGG
